# Can Conventional and Diffusion-Weighted MR Enterography Biomarkers Differentiate Inflammatory from Fibrotic Strictures in Crohn’s Disease?

**DOI:** 10.3390/medicina57030265

**Published:** 2021-03-15

**Authors:** Pietro Valerio Foti, Mario Travali, Renato Farina, Stefano Palmucci, Maria Coronella, Corrado Spatola, Lidia Puzzo, Rossella Garro, Gaetano Inserra, Gaia Riguccio, Luca Zanoli, Antonio Basile

**Affiliations:** 1 Department of Medical Surgical Sciences and Advanced Technologies “G.F. Ingrassia”—Radiology I Unit, University Hospital Policlinico “G. Rodolico-San Marco”, Via Santa Sofia, 78-95123 Catania, Italy; mario.travali.a34l@gmail.com (M.T.); radfaro@hotmail.com (R.F.); spalmucci@sirm.org (S.P.); mcoronella@sirm.org (M.C.); cor_spatola@hotmail.com (C.S.); basile.antonello73@gmail.com (A.B.); 2 Department of Medical Surgical Sciences and Advanced Technologies “G.F. Ingrassia”—Anatomic Pathology Section, University of Catania, Via Santa Sofia, 78-95123 Catania, Italy; lipizzo@unict.it (L.P.); garrorossella@gmail.com (R.G.); 3 Department of Clinical and Experimental Medicine, U.O. Medicina Interna, University of Catania, Via Santa Sofia, 78-95123 Catania, Italy; ginserra@unict.it (G.I.); gaiariguccio@gmail.com (G.R.); 4 Department of Clinical and Experimental Medicine, University of Catania, Via Santa Sofia, 78-95123 Catania, Italy; dott.zanoli@gmail.com

**Keywords:** magnetic resonance imaging, magnetic resonance enterography, diffusion weighted imaging, apparent diffusion coefficient, Crohn’s disease, fibrosis

## Abstract

*Background and Objectives:* To retrospectively assess the value of magnetic resonance enterography (MRE) parameters derived from conventional and diffusion weighted imaging (DWI) sequences to differentiate fibrotic strictures from inflammatory ones in adult patients with Crohn’s disease (CD), using surgical specimens as the histopathological reference standard. *Material and Methods*: Twenty-three patients with CD who had undergone surgical resection of ileal strictures with full-thickness histopathologic analysis within 3 months from preoperative MRE were included. Two radiologists blinded to histopathology in consensus evaluated the following biomarkers on MRE images matched to resected pathological specimens: T1 ratio, T2 ratio, enhancement pattern, mural thickness, pre-stenotic luminal diameter, and apparent diffusion coefficient (ADC). A blinded pathologist graded stricture histological specimens with acute inflammation score (AIS) and fibrosis score (FS). MRE measurements were correlated with the reference standard. *Results:* Inflammation and fibrosis coexisted in 78.3% of patients. T2 ratio was reduced in patients with severe fibrosis (*p* = 0.01). Pre-stenotic bowel dilatation positively correlated with FS (*p* = 0.002). The ADC value negatively correlated with FS (*p* < 0.001) and was different between FS grades (*p* < 0.05). The area under the receiver operating characteristic curve for discriminating between none and mild/moderate–severe bowel wall fibrosis was 0.75 for pre-stenotic bowel dilatation (sensitivity 100%, specificity 44.4%) and 0.97 for ADC (sensitivity 80%, specificity 100%). *Conclusions:* Inflammation and fibrosis often coexist in CD bowel strictures needing surgery. The combination of parameters derived from conventional MR sequences (T2 ratio, pre-stenotic dilatation) and from DWI (ADC) may provide a contribution to detect and grade bowel fibrosis in adult CD patients.

## 1. Introduction

Crohn’s disease (CD) is a chronic autoimmune systemic progressive and relapsing inflammatory disorder that can affect any part of the gastrointestinal tract. The inflammatory process can involve the whole thickness of the intestinal wall and may progress to mural fibrosis over time [1]. Both inflammation and fibrosis cause abnormal narrowing of the intestinal lumen with the formation of stenosis. Strictures, along with penetrating disease (abscesses and fistulas), are frequent complications of the disease [2]. It is estimated that within 10 years of initial diagnosis, about 70% of patients with CD develop a stricturing or perforating complication [3] and 50% need surgical intervention [4].

In particular, strictures are referred to as localized constant luminal narrowing diagnosed radiologically, endoscopically or surgically, whose functional effects may be judged from an unequivocal pre-stenotic (upstream) dilatation (i.e., ≥3 cm) [5,6,7]. Luminal narrowing is defined as bowel caliber reduction of at least 50% compared with that of a non-pathological adjacent segment [8]. Clinically, strictures lead to obstructive symptoms (abdominal pain, constipation, vomiting), thus decreasing patient’s quality of life. Histologically, strictures can be inflammatory, fibrotic or mixed [3].

Fibrotic strictures derive from an uncontrolled and excessive healing process of the intestinal wall. Owing to the transmural nature of CD, intestinal fibrosis affects all layers of the bowel wall [9]. It is characterized by extracellular matrix (ECM) protein deposition, collagen being its major component. This abnormal accumulation of ECM proteins, produced by activated myofibroblasts, results in scar formation, cytoarchitectural distortion and eventually in intestinal obstruction [3,10].

The clinical impact of noninvasively defining whether a bowel stricture is inflammatory or fibrostenotic is pivotal to correctly address the therapeutic strategy. Stenoses with an inflammatory component should undergo conservative medical management (anti-inflammatory, immunosuppressant, biological drugs). On the other hand, fibrostenotic strictures need a mechanical approach (endoscopic balloon dilation, strictureplasty or intestinal resection, on the basis of specific indications) [3,11], since currently, no specific anti-fibrotic medical therapy exists [10,12], especially for late fibrotic stages [13]. In this regard, it is important to remember that the prevalence of bowel strictures in CD patients has not significantly modified in the last two decades [10].

MRI and CT enterography are the current standards for a complete staging of the disease. Both cross-sectional imaging techniques can establish disease extension and activity, assessing mural and extramural disease [14]. Their diagnostic accuracy for the diagnosis of CD and for the detection of stenotic lesions is similar [15]; however, due to the considerable radiation burden of CT, MRI should be used when possible [6,16]. Furthermore, cine MRI sequences are able to provide functional information about bowel peristalsis [17].

Although the accuracy of cross-sectional imaging techniques for the identification of small bowel stenoses is considerable, both CT and conventional MR sequences have limited capabilities to detect and quantify fibrosis within intestinal strictures (thickened intestinal wall) [18], and therefore, to distinguish inflammatory from fibrotic stenoses.

Furthermore, unfortunately, most of the time, inflammation and fibrosis are concomitantly present to varying extents in symptomatic stenoses [3,7], and this makes it even more difficult to differentiate between predominantly inflammatory and fibrotic strictures.

In recent years, advanced MRI sequences (dynamic contrast enhanced DCE-MRI, diffusion weighted imaging DWI, intravoxel incoherent motion IVIM DWI, magnetization transfer MT MRI) [13,19,20] and hybrid imaging techniques (PET/CT, PET/MR) [18,21], able to provide quantitative parameters, have been tested in an effort to resolve this long-standing issue.

In this regard, it should be considered that the choice of the reference standard is discriminating. Indeed, endoscopic examination with mucosal biopsy can assess luminal findings and only partly the microscopic features of the disease. Conversely, the latter can be completely evaluated on an operative specimen, the only one that enables a reliable quantitative assessment of the transmural involvement of the disease [6,22].

To our knowledge, to date, only a few studies with small sample sizes have investigated the role of quantitative MR parameters using surgical specimens as the reference standard [1,13,19,20,22]; in particular, the value of DWI in detecting and, above all, in quantifying bowel fibrosis is still debated.

Tielbeek et al. [22] found that median apparent diffusion coefficient (ADC) values of fibrotic and non-fibrotic bowel segments in CD differed significantly.

In a recent study by Li et al. [19], ADC managed to differentiate fibrotic from non-fibrotic strictures, but it did not discriminate different grades of fibrosis.

In two recently published studies, IVIM DWI [13] and magnetization transfer (MT) MRI [19] outperformed traditional DWI sequences in detecting and grading bowel fibrosis.

The aim of our retrospective study was to assess the capability of qualitative and quantitative MR parameters derived from conventional MR and DWI-MR sequences to differentiate fibrotic strictures from inflammatory ones in adult patients with CD, using surgical specimens as the histopathological reference standard.

## 2. Materials and Methods

### 2.1. Patient Selection

Our institutional ethics review board approved this single-center retrospective study (AOU “Policlinico-Vittorio Emanuele” Catania, Comitato Etico Catania 1, Protocol N. 0020832, approved on 26 April 2018); informed consent was waived due to the retrospective design of the research protocol.

Using the search software of both our picture archiving and communication system (PACS) and the Anatomic Pathology Section database, we detected adult patients with CD who had undergone a magnetic resonance enterography (MRE) examination and subsequent bowel resection between January 2010 and December 2018. These patients were evaluated for inclusion in our study.

Patient inclusion criteria were as follows: age more than 18 years, diagnosis of CD established upon histological and endoscopic criteria, ileal resection within 3 months of last MRE examination and ileal stricture identified on MRE images at the location corresponding to histopathologic resected specimens.

Patient exclusion criteria were as follows: incomplete MRE protocol, nondiagnostic or poor-quality MR images due to artifacts, interval between MRE examination and surgery greater than 3 months, inability to exactly match MR findings with surgical specimens, jejunal or colon resection and prior bowel surgery.

Demographic information and indications for surgery were also extracted from patients’ electronic medical records.

### 2.2. MR Enterography Protocol

All MRE examinations were performed by using a closed configuration superconducting 1.5-T MRI unit (Signa HDxT; GE Healthcare, Milwaukee, WI, USA) with 57.2 mT/m gradient strength and 120 T/m/s slew rate, by means of an 8-channel high-resolution torso phased-array coil with array spatial sensitivity technique (ASSET) parallel acquisition. MR pulse sequences and corresponding imaging parameters of our MRE protocol are summarized in Table 1.

Patients were asked to fast for 6 h prior to the MRE examination. Bowel distension was achieved by means of per os administration of a biphasic enteric contrast agent, a polyethylene glycol (PEG) solution (Selg-esse, Alfasigma, Bologna, Italy); in particular, a total of 1000 mL was administered orally to all patients over about 45 min before the procedure. Patients underwent neither bowel cleansing, nor rectal preparation.

Patients were placed in supine (more comfortable) position on the table of the MR magnet (entry position feet first), whenever possible. In claustrophobic patients, prone position allows the examination to be carried out. MR sequences were performed during inspiratory breath hold; anatomical coverage extended from the diaphragmatic domes to through the anal canal. Scanning time was approximately 40 min.

### 2.3. Image Analysis

The evaluation of the MRE examinations was performed by two radiologists (a senior radiologist, P.V.F., with 12 years of experience in gastrointestinal MRI and a junior radiologist, M.T., with 2 years of practice) blinded to histopathological information.

Qualitative and quantitative analyses were performed by the two radiologists in consensus at the level of maximum wall thickening and luminal narrowing, by using a dedicated workstation (Advantage Windows version 4.6, General Electric Medical Systems, Milwaukee, WI, USA).

On the MR images corresponding to the resected pathological specimens (anatomical landmark: distance from ileocecal valve), qualitative and quantitative biomarkers were estimated both on conventional sequences (T1 ratio, T2 ratio, pattern of enhancement, mural thickness and maximum caliber of pre-stenotic luminal diameter) and DWI sequences (measurement of apparent diffusion coefficient).

T1 ratio was calculated using the following formula: post-contrast mural signal intensity/psoas muscle signal intensity × 100%; mural signal intensity was measured on post-contrast T1-weighted images (portal venous phase) by placing a manually drawn region of interest (ROI) in the bowel strictures.

T2 ratio was calculated through the following formula: mural signal intensity/CSF signal intensity × 100%; mural and CSF signal intensities were measured on single-shot fast spin echo (SSFSE) fat-suppressed T2-weighted images by a freehand-drawn ROI.

The pattern of enhancement was defined as homogeneous, mucosal or layered on post-contrast T1-weighted images, as previously described [22].

Mural thickness and maximum caliber of pre-stenotic upstream bowel were evaluated on fast imaging employing steady-state acquisition (FIESTA) images using calipers.

Quantitative measurement of DWI-ADC was performed on a pixel-by-pixel basis by using a dedicated diffusion analysis software (ADW 4.6 Functool; GE Healthcare). To obtain the ADC values, the radiologists placed three freehand-drawn ROIs on the DW images (b = 0, b = 800 s/mm^2^), on the thickened bowel walls, in the region of brightest signal, resulting in a total of three subreadings for each bowel segment. The images were magnified and the ROIs were defined as slightly smaller than the actual mural thickness, excluding both the bowel content and perivisceral fat in order to avoid partial volume effects. The ROIs were automatically copied to the corresponding ADC map and the ADC values were obtained according to the following formula: ADC = ln(I_b0_/I_b800_)/(b = 800 s/mm^2^ − b = 0 s/mm^2^). The corresponding T2-weighted (b = 0 s/mm^2^) images were taken into account in order to properly place the ROIs. The values of the three subreadings of each bowel segment were then averaged and the mean ADC value was calculated for each patient and used in the ensuing analysis.

### 2.4. Histologic Reference Standard and Stricture Scoring

Surgically resected ileal specimens were fixed in formaldehyde, paraffin embedded, sectioned and stained with hematoxylin-eosin as routine protocol.

All available archived hematoxylin and eosin-stained histological sections (histopathology slides) of the pertinent surgical specimens were retrieved from the archive of our institutional Anatomic Pathology Section and reviewed by an experienced digestive tract pathologist (L.P. with 30 years of experience), blinded to MRI findings. The anatomic location of the sectioned bowel segments was documented on the basis of specific anatomic landmarks (e.g., ileocecal valve or appendix).

The pathologist performed histological scoring from locations with the most severe pathological inflammation or fibrosis. In particular, inflammation was graded with an acute inflammation score (AIS) using the modified method of Borley et al. (range 0–13) [23] and fibrosis with the fibrosis score (FS) of Chiorean et al. (range 0–2) [24], as previously described [22].

### 2.5. Statistical Analysis

All tests were performed using NCSS 2007 and PASS 11 software (Gerry Hintze, Kaysville, UT, USA). The data are expressed as the mean values (and standard deviation SD) or percentages, as appropriate. MRE and histopathological variables were compared using ANOVA for continuous variables, with the Bonferroni test for multiple comparisons and the chi-squared test for categorical variables in univariate analyses. A Pearson test was used to evaluate the factors correlated with AIS. The area under the receiver operating characteristic (ROC) curve was also performed and the best cut-off was calculated as the value with the highest sensitivity and specificity. A two-tailed *p* < 0.05 was considered significant.

## 3. Results

### 3.1. Patients 

On the basis of the abovementioned criteria, 28 patients were identified for potential inclusion in the study. Of these patients, 5 had to be excluded because of the following reasons: insufficient bowel distention (n = 1), inadequate quality of MRE images undermining quantitative measurements (n = 1), difficulty in exactly matching MRE images with histologic findings of resected bowel segment (n = 1) and surgical treatment and histopathological evaluation performed at a hospital center different from ours (n = 2). Therefore, the enrolled population for the final analysis included 23 patients (14 men, 9 women) and as many (n = 23) ileal segments.

The mean age at the time of surgical treatment was 41 ± 15 years (range min–max 19–64 years). The mean number of days elapsing from preoperative MRE to surgery was 31 ± 20 days (range min–max 1–88 days).

Indications for surgical intervention included: disease refractory to pharmacological therapy and obstructive symptoms and penetrating disease.

Demographic data and medical therapy are summarized in Table 2.

### 3.2. Histopathological Assessment

Inflammation score ranged from 0 to 12 with a mean of 8.4; inflammation was graded as mild (AIS ≤ 6) in 2 cases (8.7%), moderate (AIS 7–9) in 10 cases (43.5%) and severe (AIS > 9) in 11 patients (47.8%).

FS ranged from 0 to 2 with a mean of 1; FS was graded as none (FS = 0) in 5 patients (21.7%), mild/moderate (FS = 1) in 11 cases (47.9%) and severe (FS = 2) in 7 patients (30.4%).

**Figure 1:**
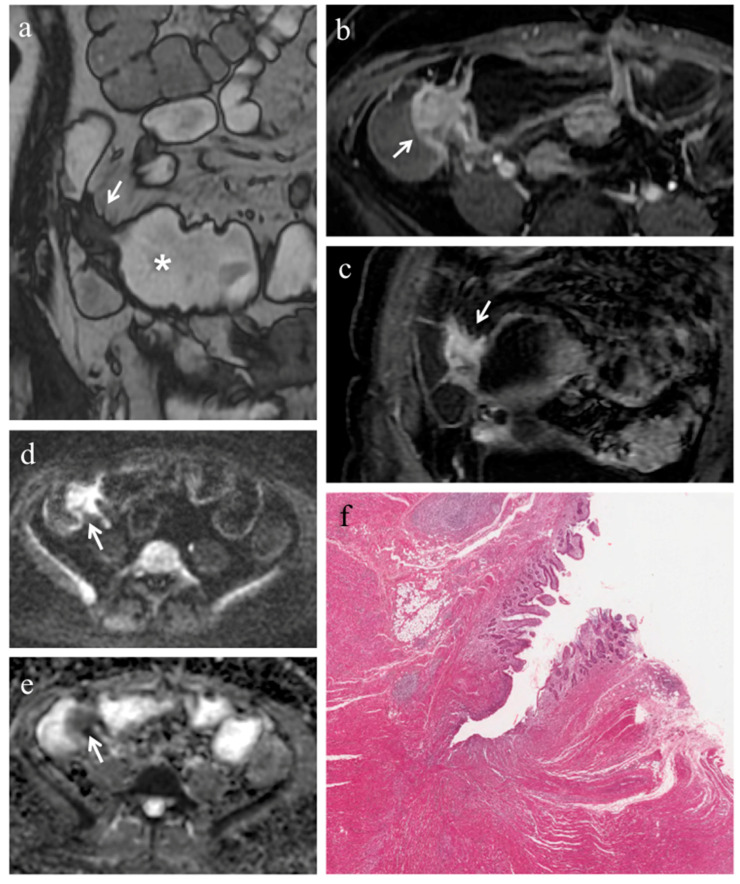
(**a**–**f**). MRE in a 49-year-old woman with CD: severe predominantly fibrotic stricture of the terminal ileum. (**a**) Coronal fast imaging employing steady state acquisition (FIESTA) image shows wall thickening with noticeable narrowing of the lumen in the terminal ileum (white arrow); note the dilatation (>30 mm) of the upstream bowel loop (white asterisk). (**b**) Axial and (**c**) coronal contrast-enhanced fat-suppressed T1-weighted images demonstrate homogeneous enhancement of terminal ileum (white arrows). (**d**) Axial DW image (b = 800 s/mm^2^) and (**e**) corresponding ADC map show the same intestinal segment demonstrating restricted diffusion with high signal intensity (white arrow) on the DWI image and low signal intensity (white arrow) on the ADC map (mean ADC value 0.745 × 10^−3^ mm^2^/s). (**f**) Histopathological section from the ileal stricture: hematoxylin and eosin-stained sample (H&E 10×). CD exhibiting severe fibrosis (FS = 2) and moderate inflammation (AIS = 7): muscular layers dissected by dense fibrotic tissue on the left, mucosal ulceration and moderate inflammatory infiltration on the right.

**Figure 2:**
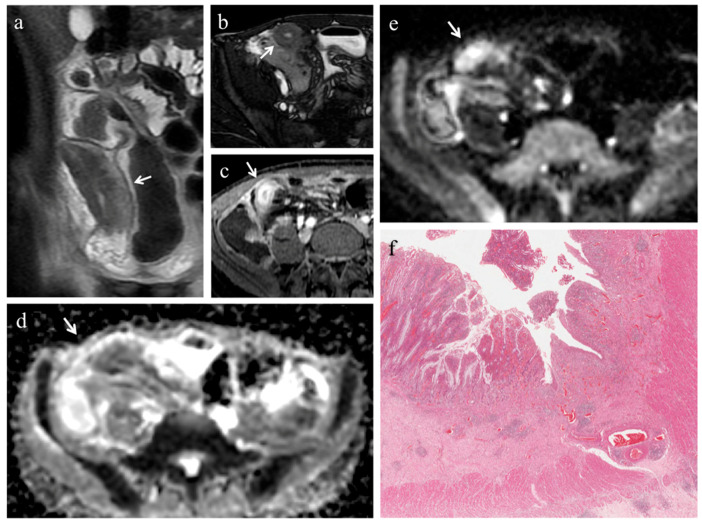
(**a**–**f**). MRE in a 19-year-old man with CD: stenosis of the terminal ileum with concomitant inflammation and fibrosis. (**a**) Coronal T2-weighted image and (**b**) axial FIESTA image show marked wall thickening of the terminal ileum with luminal narrowing (white arrows). On (**c**) axial contrast-enhanced fat-suppressed T1-weighted image, the mural thickening of the terminal ileum displays intense mucosal enhancement (white arrow). The same intestinal segment demonstrates high signal intensity on (**d**) the DW image (b = 800 s/mm^2^) and low signal intensity on (**e**) the corresponding ADC map (white arrows) (mean ADC value 1.096 × 10^−3^ mm^2^/s), a finding consistent with restricted diffusion. (**f**) Histopathological section from the ileal stricture: hematoxylin and eosin-stained sample (H&E 10×). CD exhibiting mild/moderate fibrosis (FS = 1) and severe inflammation (AIS = 10): mucosal ulceration and severe inflammatory infiltration on the top; mild/moderate fibrosis, edema and inflammatory infiltration in submucosal layer on the bottom.

**Figure 3:**
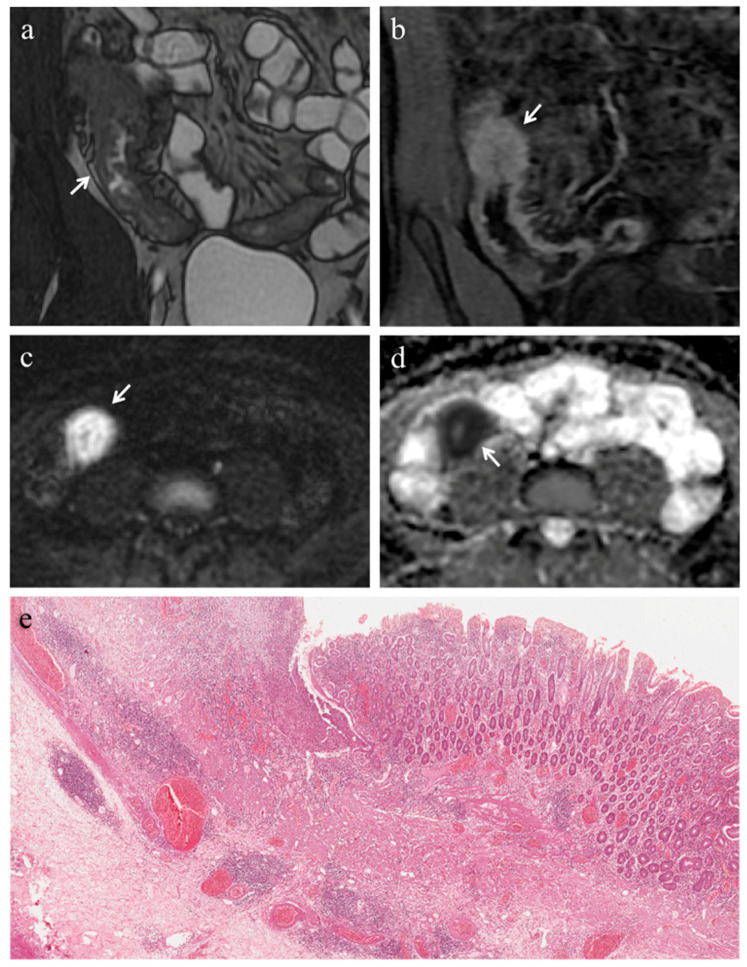
(**a**–**e**). MRE in a 22-year-old man with CD: predominantly inflammatory stricture of the terminal ileum. (**a**) Coronal FIESTA image shows mural thickening of terminal ileum with stenosis of the lumen (white arrow). (**b**) Coronal contrast-enhanced fat-suppressed T1-weighted image demonstrates the homogeneous wall enhancement of the affected ileal loop. The same intestinal segment demonstrates restricted diffusion in the form of high signal intensity on (**c**) the DW image (b = 800 s/mm^2^) and low signal intensity on (**d**) the corresponding ADC map (white arrows) (mean ADC value 1.320 × 10^−3^ mm^2^/s). (**e**) Histopathological section from the ileal stricture: hematoxylin and eosin-stained sample (H&E 10×). CD exhibiting absent or minimal fibrosis (FS = 0) and severe inflammation (AIS = 9): mucosal ulceration and intense inflammatory infiltration on the top; edema and intense inflammatory infiltration in the submucosal layer.

In 18 (78.3%) ileal segments, inflammation and fibrosis coexisted (Figure 1 and Figure 2), in 5 (21.7%) cases inflammatory alterations without fibrosis were found (Figure 3), whereas under no circumstances was fibrosis without inflammation encountered. No significant correlation was highlighted between AIS and FS (*p* = 0.22).

### 3.3. Analysis of Conventional MRE Sequences

T1 ratio and T2 ratio. The T1 ratio did not show any correlation with FS (*p* = 0.56). There was a trend towards a decrease in the T1 ratio when AIS was higher, although this was not significant (r = −0.319, *p* = 0.14). The T2 ratio was reduced in patients with severe fibrosis (0.39 ± 0.12 vs. 0.26 ± 0.06; *p* = 0.01), compared to those with none or mild/moderate fibrosis. A trend towards an increase in T2 ratio when AIS was higher was observed, although this did not reach the significance level (r = 0.396, *p* = 0.06).

Pattern of enhancement. The diseased bowel wall showed the following enhancement patterns: homogeneous in 6 cases (26.1%) (Figure 1b,c, Figure 3b), mucosal in 8 cases (34.8%) (Figure 2c) and layered in 9 cases (39.1%). Pattern of enhancement did not show significant variations according to AIS (*p* = 0.56) or FS (*p* = 0.49).

Mural thickness. Mural thickness (mean 10 ± 2 mm, range 6–14 mm) was not associated with inflammation score (*p* = 0.84) or fibrosis (*p* = 0.96).

**Figure 4:**
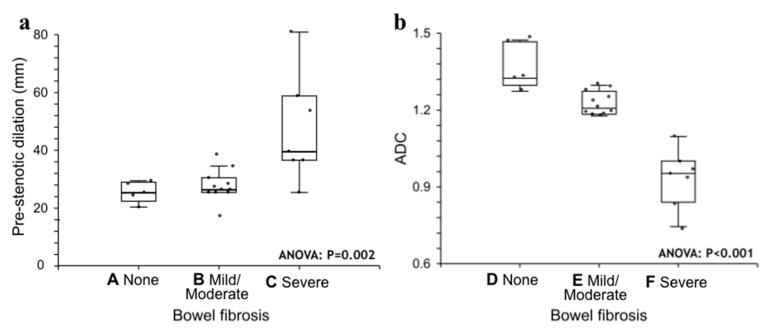
(**a**,**b**). Statistical analysis. Pre-stenotic upstream bowel dilatation (**panel a**) and ADC (**panel b**) sorted by fibrosis score. Medians are given with the interquartile range (IQR). ANOVA tests were performed. Bonferroni multiple comparison test: A ≠ C; B ≠ C; D ≠ E ≠ F. ADC = apparent diffusion coefficient.

**Figure 5:**
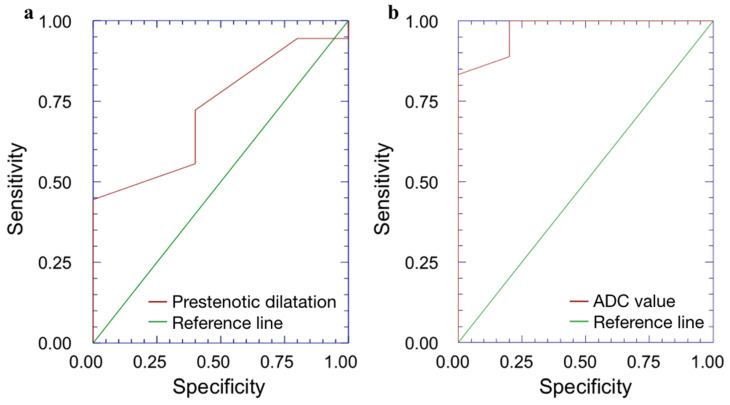
(**a**,**b**). Receiver operating characteristic (ROC) analysis. (**a**) The ROC curve shows that pre-stenotic upstream bowel dilatation has a high accuracy with AUC 0.75 (*p* < 0.001) for discriminating between none and mild/moderate–severe bowel wall fibrosis. (**b**) The ROC curve demonstrates that ADC shows a high accuracy with AUC 0.97 (*p* < 0.001) for discriminating between none and mild/moderate–severe bowel wall fibrosis. ADC = apparent diffusion coefficient, AUC = area under ROC curve.

Pre-stenotic luminal diameter. Maximum caliber of pre-stenotic upstream bowel (mean 33 ± 14 mm, range 17–80 mm) did not correlate with inflammation score (*p* = 0.50). On the other hand, pre-stenotic bowel dilatation positively correlated with the fibrosis score (*p* = 0.002): upstream bowel dilatation increased according to fibrosis grade (Figure 1a). In particular, pre-stenotic luminal diameter was higher in patients with severe fibrosis than in those with none or mild/moderate fibrosis (*p* < 0.05) (Figure 4, Panel a). The mean pre-stenotic luminal diameters were 25 ± 4 mm, 27 ± 5 mm and 47 ± 18 mm for none, mild/moderate and severe FS, respectively. Moreover, this measurement showed a high accuracy (AUC 0.75, *p* < 0.001) for discriminating between none and mild/moderate–severe bowel wall fibrosis; using a threshold value of 30 mm, the sensitivity and specificity were 100% and 44%, respectively (Figure 5, Panel a).

### 3.4. Analysis of DWI Quantitative Measures (ADC)

The mean size of the ROIs used to measure the ADC value in the diseased bowel wall was 48 ± 22 mm^2^. The overall mean of ADC values was 1.168 ± 0.181 × 10^−3^ mm^2^/s (range 0.745–1.473 × 10^−3^ mm^2^/s). No significant correlation was found between ADC and inflammation score (*p* = 0.41). The ADC value was negatively correlated with the fibrosis score (*p* < 0.001). The ADC value was different between FS grades (*p* < 0.05) (Figure 4, Panel b). The means of the ADC values were 1.371 × 10^−3^ mm^2^/s for none, 1.224 × 10^−3^ mm^2^/s for mild/moderate and 0.936 × 10^−3^ mm^2^/s for severe FS. ADC values showed a high accuracy (AUC 0.97, *p* < 0.001) for discriminating between none and mild/moderate–severe bowel wall fibrosis. Using a threshold ADC value of 1.300 × 10^−3^ mm^2^/s, the sensitivity and specificity were 80% and 100%, respectively (Figure 5, Panel b). Qualitative and quantitative MRE findings are summarized in Table 3.

## 4. Discussion

In our study, we tested various MR measurements derived from both conventional MR and DWI-MR sequences in an attempt to differentiate fibrotic from inflammatory strictures in adult patients with CD. Our study demonstrated that pre-stenotic dilatation, T2 ratio and mean ADC value correlated (positively for the former and negatively for the latter two) with bowel wall fibrosis in adult patients with CD.

In agreement with previous reports [1,18,19,20], we found that, even if without a statistically significant correlation, pathologically inflammation and fibrosis almost always coexist in CD bowel strictures needing surgery; this leads to a difficulty by conventional imaging techniques to detect and gauge fibrosis within intestinal strictures. As proof of this, as previously experienced by other authors [1], most of the qualitative and quantitative conventional parameters we analyzed failed to significantly correlate with inflammation or fibrosis.

Among the conventional MRE findings we assessed, pre-stenotic dilatation significantly positively correlated with fibrosis. This result is consistent with another previous retrospective study conducted on 20 pediatric CD patients by Barkmeier et al. [1] in which the authors found strictures with >3 cm upstream dilatation being highly associated with transmural fibrosis. According to us this finding could be explained by the lower stretch of fibrostenotic strictures, due to scar tissue formation, compared to inflammatory ones; as a consequence, the former strictures are more likely to cause greater dilatation of the upstream bowel loops.

In our study, another quantitative conventional parameter, manifestation of intrinsic MR tissue contrast, the T2 ratio, was reduced in patients with severe fibrosis, compared to those with no or mild/moderate fibrosis. Moreover, there was a trend towards an increase in T2 ratio and a rise in inflammation score, but without reaching statistical significance. This finding is consistent with that of the study of Tielbeek et al. [22], in which the T2 mural/CSF ratio was able to discriminate fibrosis from inflammation. Therefore, strictures characterized by severe fibrosis should generally show low signal intensity on T2-weighted images, unless severely inflamed. Indeed, unlike our study and that of Tielbeek et al. [22], Barkmeier et al. [1] found no correlation between normalized T2 signal intensity and fibrosis score, probably due to the presence of superimposed severe inflammation, and therefore edema, within strictures with transmural fibrosis.

In recent years, the traditional assumption that intestinal fibrosis in CD is an irreversible process is progressively modifying into a more dynamic view, according to which fibrosis is a chronic and progressive process, mediated by complex genic, molecular and cell interactions, which could be reversible, at least at an early phase of the disease course [10].

These recent acquisitions concerning the pathogenesis of fibrosis has given impetus for the search for new anti-fibrotic agents, whose effectiveness has still to be validated. In this setting, novel imaging biomarkers (such as radiation-free quantitative DW MRI-based parameters) that could noninvasively, accurately and reliably quantify fibrosis and monitor the evolution of the fibrotic process, thus overcoming the limits of conventional imaging techniques, are needed.

In our study, the mean ADC value, the quantitative parameter of DWI, negatively correlated with bowel wall fibrosis and demonstrated significant differences between degrees of fibrosis. This result is consistent with the ones of recently published articles. Tielbeek et al. [22] found that a decrease in ADC values significantly correlated with fibrosis score. In a retrospective study, Catalano et al. demonstrated a hybrid imaging biomarker derived from PET/MRE, ADC x SUVmax, showing significantly lower values in the fibrosis-only histologic group compared with the mixed fibrosis and active inflammation or active inflammation-only groups.

The reason for the decrease of ADC values in fibrotic bowel strictures is already known. Abnormal deposition of collagen in the ECM, which characterizes intestinal fibrosis in CD, causes a narrowing of the extracellular space and decreases the motion of water molecules, namely, the diffusivity of water. These cellular and molecular changes translate into a reduction of ADC [13,20].

Nevertheless, the results about the role of DWI-ADC are not unambiguous. In a retrospective study conducted by Barkmeier et al. [1], no significant correlation between ADC and histological fibrosis or inflammation scores was found in pediatric CD patients.

The cause of these discordant results can be partially explained by the limits of traditional DWI sequences, as recently revealed by some authors [13,19,20]. In a study by Li et al. [19], the relationship between normalized MT ratio and bowel fibrosis was not affected by the amount of inflammation. On the other hand, in another study, the same authors [20] found that the capability of ADC to assess intestinal fibrosis decreased with increasing degrees of bowel inflammation. These results demonstrate that MT is a more robust and reliable tool than ADC in detecting and grading bowel fibrosis. In two recently published studies, magnetization transfer (MT) [19] and another MRI-based quantitative parameter, fractional perfusion, derived from intravoxel incoherent motion (IVIM) DWI [13], outperformed both ADC value and contrast-enhanced (CE) imaging in detecting and grading intestinal fibrosis in adult patients with CD. Therefore, the authors argued that in traditional DWI sequences, the superimposition of diffusion and perfusion phenomena may impair the specificity of the technique [13].

Moreover, the way to perform quantitative measurements should also be taken into account. In agreement with previous authors [13,18,19,20], to obtain the ADC values of the abnormal bowel walls, we placed three ROIs slightly smaller than the actual mural thickness, excluding the bowel content and perivisceral fat. Nevertheless, unlike Catalano et al. [18], who employed an oval ROI, we shaped a freehand-drawn ROI in order to make our measurements as precise as possible.

Despite the abovementioned limitations, traditional DWI has various strengths. It enables a dual evaluation: qualitative, based on visual assessment of native DW images (in particular with a high *b* value), and quantitative, with measurement of the ADC value on corresponding maps. Furthermore, it is radiation-free, easy to run, even without the supervision of a radiologist, and its post-processing is comparatively simple and not time consuming. This last aspect is of paramount importance, since translating quantitative MR imaging techniques into daily clinical practice requires making them easy to perform and to interpret [25].

Interestingly, in our study, the conventional MRE parameter, pre-stenotic dilatation (cutoff of ≥30 mm) was associated with a very high sensitivity (100%) and a quite low specificity (44.4%) for discriminating between none and mild/moderate–severe bowel wall fibrosis; on the other hand, the DWI derived quantitative parameter ADC (cutoff of ≤1.30 × 10^−3^ mm^2^/s) showed a very high specificity (100%) and a lower sensitivity (80%) for the same task. These results suggest that the association of the two abovementioned parameters could represent a winning combination to distinguish fibrotic and non-fibrotic strictures. In particular, to perform an ADC measurement, above all in patients demonstrating a caliber of pre-stenotic upstream bowel ≥ 30 mm, would accurately identify fibrotic strictures in daily clinical activity, practically and without requiring a vast amount of time.

The main strength of our work is the full-thickness histopathologic analysis of surgical specimens we used as a reference standard. Additionally, we included only ileal lesions in order to make the patient population more homogeneous.

At the same time, however, in our study, a number of limitations need to be considered. The first limitation is the retrospective design of the study. This may have hindered, to some extent, the correct point-by-point correlation (location matching) between MR images and surgical specimens. However, in order to limit the possibility of bias, unlike recent prospective reports [13,19,20,22], we analyzed only one ileal segment per patient. Moreover, unlike previous retrospective studies [1,26], in which MR systems with different field strengths (1.5- and 3.0-tesla) were used, our MRI scanner remained the same for the entire duration of the study and the MRE imaging protocol did not undergo modifications, DWI sequences being present from the beginning. The second limitation, the small number of patients, is due to our reference standard, full-thickness histopathological specimens, closely linked to a limited subgroup of patients needing surgical intervention. Lastly, owing to the retrospective nature of the study, the interval between preoperative MRE and surgical intervention (average 35 days, range 1–88 days) is longer than that of other recent prospective studies (15 days) [13,20]. Nevertheless, it is also true that in a timeframe such as the aforementioned one we would not expect to find considerable changes in the amount of fibrosis within the intestinal wall.

## 5. Conclusions

Our study demonstrated that pre-stenotic dilatation, T2 ratio and mean ADC value correlated with bowel wall fibrosis in adult CD patients undergoing surgery. Nevertheless, because of the almost constant coexistence of fibrosis and inflammation within intestinal stenoses of symptomatic CD patients, no single parameter is able to accurately distinguish predominantly inflammatory from fibrotic strictures. In our opinion, the combination of parameters obtained from conventional MR sequences, allowing morphological characterization (luminal narrowing, longitudinal extent of the affected intestine, amount of pre-stenotic dilatation), and quantitative measures derived from DWI sequences (ADC) may provide a contribution to detect and grade bowel fibrosis in adult CD patients.

We know that there are different phenotypes of CD and that some patients may present a more rapid evolution towards intestinal fibrosis than others [1,7,26]. Therefore, in our opinion, future research should focus on the application of “omic” sciences. In particular, radiomics and radiogenomics, through a better understanding of disease biology, might more accurately depict tissue heterogeneity of layered bowel wall strictures, in which inflammation and fibrosis coexist, and may provide new information to develop imaging biomarkers reflecting genotypic and phenotypic features of pathologic alterations. The novel molecular understanding of CD and the proper use of advanced imaging techniques may allow to quantify signal modifications, expression of underlying tissue microenvironment, thus going beyond morphologic features in the direction of individualized therapeutic approaches.

This scenario enhances even further the radiologist’s role as an integral part of the multidisciplinary team, essential to diagnose and follow-up CD patients and to correctly guide the therapeutic decision-making process.

## Figures and Tables

**Table 1:** MRE protocol. Synoptic table summarizes the scanning parameters of MR pulse sequences. Coronal T1-weighted 3D gradient-echo liver acquisition with volume acceleration (LAVA) sequences with fat suppression were acquired before and after intravenous administration of paramagnetic contrast material (0.1 mmol/kg, followed by 20 mL of saline solution, both at a flow rate of 2 mL/s); in particular, the sequence was acquired at 60 and 120 s after contrast administration. An anti-peristaltic agent, scopolamine-N-butyl bromide (Buscopan^®^ 20 mg; Boehringer Ingelheim, Milano, Italy), was administered intravenously just before post-contrast imaging to reduce peristaltic artifacts.

MRI Protocol	Coronal T2W SSFSE	Coronal FIESTA	Coronal T2W Thick-slab SSFSE	Axial DWI SE EPI	Sagittal, Coronal, Axial T1W 3D GRE LAVA
Repetition time/Echo time (ms)	705/90	4/1.7	2408/1103	3000/74	4.1/1.9
Flip angle	90°	75°	90°	90°	12°
Section Thickness (mm)	6	6	70	8	3.4
Interslice gap (mm)	0.6	0.6	—	2	1.7
Bandwidth (kHz)	83.33	100	31.25	250	62.5
Field of view (cm)	44–48	44–48	44–48	42	44–48
Matrix	384 × 224	512 × 384	512 × 384	160 × 160	320 × 192
N. of signal acquired	0.57	1	1	2	0.7
N. of images	28	28	1	15	120
Frequency direction	Right to left	Right to left	Right to left	Anterior to posterior	Superior to inferior
Acquisition time (s)	24	22	2	27	23
B-value (s/mm^2^)	—	—	—	0–800	—

T2W = T2-weighted, T1W = T1-weighted, SSFSE = single-shot fast spin-echo, FIESTA = fast imaging employing steady-state acquisition, DWI = diffusion-weighted imaging, SE = spin-echo, EPI = echoplanar imaging, GRE = gradient-echo, LAVA = liver acquisition with volume acceleration.

**Table 2:** Demographic data and medical therapy.

Patient	Gender	Age	Smoker	Disease Duration	Therapy
Case 1	M	32	former	10 years 5 months	5-ASA
Case 2	M	30	active	8 years 4 months	CS, IFX, ADA
Case 3	F	51	no	8 months	5-ASA, CS
Case 4	M	45	active	1 years 5 months	5-ASA, CS
Case 5	F	61	active	25 years 7 months	CS, IFX, ADA
Case 6	M	64	former	33 years	5-ASA, CS
Case 7	M	36	active	5 years 9 months	5-ASA
Case 8	M	58	no	11 years 5 months	5-ASA, CS
Case 9	M	52	active	7 months	none
Case 10	F	48	former	1 years 4 months	5-ASA, CS
Case 11	M	56	no	12 years 3 months	5-ASA, CS
Case 12	F	35	no	5 years 10 months	5-ASA, CS, ADA
Case 13	M	32	no	7 years 2 months	CS, IFX
Case 14	F	49	former	21 years 7 months	5-ASA, IFX
Case 15	F	22	no	7 years 2 months	5-ASA, AZA
Case 16	F	62	former	1 years 10 months	5-ASA, CS
Case 17	F	63	former	1 years 6 months	none
Case 18	M	19	no	3 months	5-ASA, CS
Case 19	M	33	active	6 years 2 months	5-ASA, AZA
Case 20	F	20	no	11 months	5-ASA, CS
Case 21	M	22	former	1 years	5-ASA, IFX
Case 22	M	33	active	4 years 7 months	CS
Case 23	M	32	active	5 years 10 months	5-ASA, CS, AZA

5-ASA = Mesalazine; CS = Corticosteroid; IFX = Infliximab; ADA = Adalimumab; AZA = Azathioprine.

**Table 3:** Qualitative and quantitative MR enterography findings of bowel strictures.

Thickness (mm)	Luminal Diameter (mm)	T1 Ratio	T2 Ratio	Pattern of Enhancement	ADC × 10^−3^ mm^2^/s
11	25	1.928	0.291	layered	1.473
11	36	2.342	0.283	homogeneous	1.46
8	38	2.012	0.201	mucosal	1.32
9	26	1.958	0.346	mucosal	1.327
9	36	1.897	0.263	layered	1.273
9	80	2.062	0.138	homogeneous	1.247
10	25	1.943	0.575	mucosal	1.177
12	30	1.857	0.307	layered	1.19
6	24	1.563	0.306	homogeneous	1.18
10	39	2.464	0.268	mucosal	1.21
10	20	2.059	0.365	layered	1.233
8	27	2.263	0.591	layered	1.287
11	17	1.75	0.388	layered	1.297
9	58	2.305	0.213	homogeneous	1.183
9	25	1.858	0.333	layered	1.193
8	28	1.938	0.541	mucosal	1.273
10	25	2.043	0.537	layered	0.94
10	53	1.876	0.349	mucosal	0.84
10	28	2.828	0.245	mucosal	0.745
12	26	2.222	0.266	layered	0.955
14	29	1.782	0.368	homogeneous	1.001
9	34	1.768	0.365	homogeneous	0.972
9	25	2.002	0.474	mucosal	1.097

ADC = apparent diffusion coefficient.

## Data Availability

The data presented in this study are available on request from the corresponding author.

## Abbreviations

ADC	Apparent diffusion coefficient
AIS	Acute inflammation score
ASSET	Array spatial sensitivity technique
AUC	Area under the curve
CD	Crohn’s disease
CE	Contrast-enhanced
CT	Computed tomography
DCE	Dynamic contrast enhanced
DWI	Diffusion weighted imaging
ECM	Extracellular matrix
FOV	Field of view
FS	Fibrosis score
IVIM	Intravoxel incoherent motion
MRE	Magnetic resonance enterography
MRI	Magnetic resonance imaging
MT	Magnetization transfer
PACS	Picture archiving and communication system
ROC	Receiver operating characteristic
ROI	Region of interest
SD	Standard deviation
